# Increased Thromboxane A_2_ Levels in Pulmonary Artery Smooth Muscle Cells Isolated from Patients with Chronic Obstructive Pulmonary Disease

**DOI:** 10.3390/medicina59010165

**Published:** 2023-01-13

**Authors:** Abdullah A. Alqarni

**Affiliations:** Department of Respiratory Therapy, Faculty of Medical Rehabilitation Sciences, King Abdulaziz University, Jeddah 22230, Saudi Arabia; aaalqarni1@kau.edu.sa

**Keywords:** COPD, pulmonary artery smooth muscle cells, thromboxane A_2_, pulmonary hypertension

## Abstract

*Introduction:* Pulmonary hypertension due to chronic obstructive pulmonary disease (COPD) is classified as Group 3 pulmonary hypertension, with no current proven targeted therapies. It has been shown that cigarette smoke, the main risk factor for COPD, can increase thromboxane A_2_ production in healthy human pulmonary artery smooth muscle cells and pulmonary artery endothelial cells, and that blocking the effect of increased thromboxane A_2_ using daltroban, a thromboxane A_2_ receptor antagonist, can inhibit cigarette smoke-induced pulmonary artery cell proliferation. However, it is largely unknown whether thromboxane A_2_ is increased in smokers with COPD. Therefore, the aim of this study was to assess the level of thromboxane A_2_ production in patients with COPD who smoke. *Methods:* Pulmonary artery smooth muscle cells from three smokers with COPD and three healthy donors were cultured in cell culture medium. The culture medium was collected and the thromboxane B_2_ (a stable metabolite of thromboxane A_2_) released in the culture medium was quantified using an ELISA kit. The data were normalised with the total protein concentration and then expressed in pg/mg protein. Demographic data were collected and baseline pulmonary function tests of patients with COPD were conducted. *Results:* The mean age of patients with COPD was 69 ± 7 years. All patients were smokers and had a mean smoking history of 39.66 ± 9.50 packs per year. The mean forced expiratory volume in one second, that is, FEV1%, and the ratio of forced vital capacity (FVC) to FEV1% of COPD patients were 63.33 ± 19.60% and 52.66 ± 14.64%, respectively. The results revealed that thromboxane A_2_ production was significantly increased in pulmonary artery smooth muscle cells from smokers with COPD (434.56 ± 82.88 pg/mg protein) compared with the thromboxane _A2_ levels in pulmonary artery smooth muscle cells from healthy donors (160 ± 59.3 pg/mg protein). *Conclusions:* This is the first report of increased thromboxane A_2_ production in pulmonary artery smooth muscle cells from smokers with COPD. This observation strongly suggests that thromboxane A_2_ can be used as a novel therapeutic target for the treatment of patients with COPD-associated pulmonary hypertension.

## 1. Introduction

Pulmonary hypertension is a fatal disease clinically defined by increased mean pulmonary arterial pressure and a mean pulmonary arterial pressure (mPAP) greater than 20 mmHg at rest, based on haemodynamic assessments obtained using right heart catheterisation [1]. Pulmonary hypertension is a pathophysiological disorder that involves several clinical conditions associated with different cardiovascular and respiratory diseases. Accordingly, pulmonary hypertension has been classified into five groups: pulmonary arterial hypertension (Group 1), pulmonary hypertension due to left heart disease (Group 2), pulmonary hypertension due to lung diseases and/or hypoxia (Group 3), pulmonary hypertension due to pulmonary artery obstructions (Group 4) and pulmonary hypertension due to unclear mechanisms (Group 5) [1].

Group 2 pulmonary hypertension is the second-most-common form of pulmonary hypertension and is more prevalent in patients with chronic pulmonary lung disease (COPD) than any other chronic pulmonary disease [1]. According to a global initiative for chronic obstructive lung disease, pulmonary hypertension is observed in up to 90% of patients with COPD. Cigarette smoke, the most prominent risk factor for COPD, has been shown to induce pulmonary vascular remodelling, which leads to increased pulmonary vascular resistance, pulmonary arterial pressure and, ultimately, pulmonary hypertension [2,3,4]. Findings reported by our group and others revealed that cigarette smoke extract could induce the proliferation of pulmonary artery cells, including pulmonary artery smooth muscle cells [5,6,7]. Although the underlying mechanism is not completely understood, our previous findings strongly suggest that imbalanced prostanoids play a key role in the cigarette smoke-induced proliferation of pulmonary artery cells [5].

Prostanoids, consisting of prostaglandins and thromboxanes, are major metabolites of arachidonic acid. To produce prostanoids, rate-limiting prostaglandin H2 synthase (cyclooxygenase) converts arachidonic acid into unstable prostaglandin H2. Prostaglandin H2, by specific synthases and isomerases, is then converted to prostanoids: thromboxane A_2_, prostaglandin E2, prostaglandin I2, prostaglandin F2α and prostaglandin D2. Among these prostanoids, early evidence suggests that thromboxane A_2_ and prostaglandin I2 (also called prostacyclin) are critically involved in the development of pulmonary hypertension. Thromboxane A_2_ is known to promote proliferation and vasoconstriction, whereas prostaglandin I2 has opposing effects by inducing anti-proliferation and vasodilation on the pulmonary vasculature. An imbalance between thromboxane A_2_ (increased) and prostaglandin I2 (decreased) has been reported in urinary excretions obtained from patients with pulmonary hypertension [8]. Although the levels of thromboxane A_2_ have not previously been assessed in patients with COPD, a previous study showed that PGI2 production is reduced in the lungs of smokers with COPD [9]. More importantly, the prostaglandin I2 analogue inhaled treprostinil, the first approved drug for patients with Group 3 pulmonary hypertension, was approved in May 2022 for the treatment of patients with interstitial lung disease-associated pulmonary hypertension [1]. As there are no therapies currently approved for the treatment of patients with COPD-associated pulmonary hypertension, rigorous studies are needed to enhance our understanding of the role of cigarette smoke-induced pulmonary vascular remodelling in patients with COPD.

It has been demonstrated that cigarette smoke, the strongest risk factor for COPD, can induce imbalanced prostanoids, characterised mainly by increased thromboxane A_2_ production in healthy human pulmonary artery smooth muscle cells and pulmonary artery endothelial cells [5]. More importantly, it has also been reported that blocking the effect of increased thromboxane A_2_ using daltroban, a thromboxane A_2_ receptor antagonist, can inhibit the cigarette smoke-induced proliferation of pulmonary artery smooth muscle cells and pulmonary artery endothelial cells [5]. However, it is largely unknown whether thromboxane A_2_ is greater in pulmonary artery cells from smokers with COPD. Therefore, this study aimed to explore the levels of thromboxane A_2_ production in pulmonary artery smooth muscle cells obtained from patients with COPD who smoke.

## 2. Materials and Methods

### 2.1. Pulmonary Artery Smooth Muscle Cell Culture

Human pulmonary artery smooth muscle cells from three patients with COPD and from three healthy donors were used in the current study. Cells from healthy donors were obtained as previously described [5] from Thermo Fisher Scientific, Waltham, MA, USA, Cell Applications, San Diego, CA, USA, and Lonza Group, Basel, Switzerland, with no history of respiratory disease or evidence of pulmonary vascular abnormalities. Human pulmonary artery smooth muscle cells from three smokers with a confirmed diagnosis of COPD (Table 1) were supplied by Prof Linhua Pang and Dr Rachel L. Clifford (University of Nottingham, Nottingham, UK). Both healthy and diseased cells were received at passage 3, sub-cultured in Dulbecco’s Modified Eagles Medium (DMEM) with 20% foetal bovine serum (FBS) as previously described [5] and used at passage 6. Pulmonary artery smooth muscle cells were cultured in a six-well plate with DMEM with 20% FBS for 48 h until the cells reached 100% confluence. The cells were then serum starved in DMEM with 0.5% FBS for the last 24 h, as previously described [5]. Protein and cell culture medium were collected at the end of a total of 72 h to measure the protein concentration and mediator release thromboxane B_2_, respectively.

### 2.2. Thromboxane B_2_ Analysis

At the end of the incubation time, the culture medium was collected and the thromboxane B_2_ (stable metabolite of thromboxane A_2_) that had been released in the culture medium was then measured using an ELISA kit. The kits were obtained from Cayman Chemical and thromboxane B_2_ analysis was performed following the manufacturer’s instructions. A bicinchoninic acid assay kit was obtained from Thermo Fisher Scientific and used to quantify the protein concentrations following the manufacturer’s instructions. The data were normalised with the total protein concentration and then expressed in pg/mg protein. 

### 2.3. Statistical Analysis

In this study, GraphPad Prism 9 was used for statistical analysis. In order to determine the significant differences between the means, an unpaired two-tailed Student’s *t*-test was used. *p* values of <0.05 were accepted as statistically significant.

## 3. Results

It has previously been shown that cigarette smoke extract can increase the levels of thromboxane A_2_ in both pulmonary artery smooth muscle cells and PAECs and that the use of a thromboxane A_2_ receptor antagonist can inhibit cigarette smoke extract-induced pulmonary artery smooth muscle cell and pulmonary artery endothelial cell proliferation. These observations strongly suggest that blocking the effects of thromboxane A_2_ can reduce the pulmonary vascular remodelling induced by cigarette smoke. To strengthen these previous findings, I wanted to determine the levels of thromboxane A_2_ in pulmonary artery smooth muscle cells isolated from patients with COPD. In particular, it was of interest to find out whether cells from COPD patients who smoke can produce higher levels of thromboxane A_2_ than that seen in healthy donors.

The demographics and clinical characteristics of the COPD patients included in this study are reported in Table 1. The mean age of the COPD patients was 69.6 ± 7.37 years. The COPD patients had a mean smoking history of 39.66 ± 9.50 packs per year. The mean forced expiratory volume in one second (FEV1), FEV1%, forced vital capacity (FVC) and FVC/FEV1% of COPD patients were 1.9 ± 0.57 L, 63.33 ± 19.60%, 3.79 ± 0.80 L and 52.66 ± 14.64%, respectively (Table 1).

**Figure 1:**
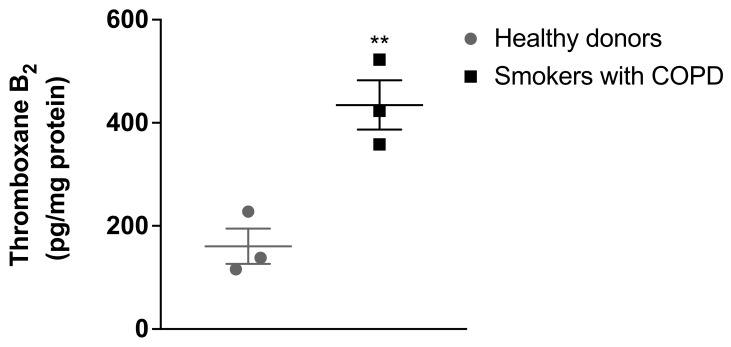
Levels of thromboxane B_2_ in pulmonary artery smooth muscle cells from COPD patients who smoke. Confluent human pulmonary artery smooth muscle cells from healthy donors and patients with COPD with a history of smoking were placed in a cell culture medium for 72 h. The medium was collected and the levels of thromboxane B_2_ were determined by ELISA. The results were normalised with total cell protein and expressed in pg/mg protein. Each data point represents the mean ± SEM using pulmonary artery smooth muscle cells from three healthy donors or smokers with COPD. ** *p* < 0.01 compared with pulmonary artery smooth muscle cells isolated from healthy donors.

As illustrated in Figure 1, the results demonstrated that pulmonary artery smooth muscle cells from healthy donors produced thromboxane A_2_, measured as TXB_2_ (160 ± 59.3 pg/mg protein). The vasoconstrictor and proliferative thromboxane A_2_ levels were found to be significantly increased in the pulmonary artery smooth muscle cells isolated from patients with COPD who smoke (434.56 ± 82.88 pg/mg protein) compared with the thromboxane A2 levels in pulmonary artery smooth muscle cells isolated from healthy donors.

## 4. Discussion

To the best of the author’s knowledge, this is the first report of increased thromboxane A_2_ in pulmonary artery smooth muscle cells isolated from COPD patients who smoke. This supports our previous findings that thromboxane A_2_ is increased in healthy pulmonary artery smooth muscle and endothelial cells treated with cigarette smoke and that blocking the effects of increased thromboxane A_2_ using a thromboxane receptor antagonist can inhibit cigarette smoke-induced pulmonary artery smooth muscle and endothelial cell proliferation. These observations, together with the findings presented in this study, suggest that targeting the prostanoid pathway, particularly thromboxane A_2_, may serve as a potential novel therapeutic option to treat patients with COPD-associated pulmonary hypertension. 

Nitric oxide, prostanoid and endothelin are the three pathways currently used to treat patients with pulmonary hypertension [10]. Despite advances in the diagnosis and treatment of pulmonary hypertension, several conditions within the groups of pulmonary hypertension remain without approved therapies, one of which is COPD associated with pulmonary hypertension [1]. Patients with pulmonary hypertension due to COPD are treated and managed with either systematic vasodilators or specific therapies approved for Group 1 or Group 3 pulmonary hypertension [1]. Among the three known pathways, targeting prostanoids has been proven effective in the treatment of patients with Group 3 pulmonary hypertension, particularly pulmonary hypertension due to interstitial lung disease (ILD) [1]. Recently, the U.S. Food and Drug Administration approved inhaled treprostinil, a prostaglandin I2 analogue, following a randomised controlled clinical trial that revealed enhanced exercise capacity [11].

With prostaglandin I2, thromboxane A_2_ plays a key role in regulating cardiovascular homeostasis. It induces pulmonary vasoconstriction, pulmonary artery cell proliferation and platelet aggregation [10]. An imbalance with increased thromboxane A_2_ and reduced prostaglandin I2 is thought to contribute to pulmonary hypertension [8]. In support, our research group previously found that cigarette smoke can induce an imbalance between thromboxane A_2_ and prostaglandin I2 production in pulmonary artery smooth muscle and endothelial cells, mainly due to increased thromboxane A_2_ [5]. This supports the finding presented in the current study that thromboxane A_2_ is increased in the pulmonary artery smooth muscle cells of patients with COPD and suggests a key role of pulmonary artery smooth muscle cells in the development of pulmonary hypertension. In addition, these observations suggest that pulmonary artery smooth muscle cells can be among the targets of therapies for pulmonary hypertension, as they have emerged as key contributors not only to pulmonary vasoconstriction, but also to pulmonary vascular remodelling [12].

Given the detrimental effects of thromboxane A_2_ in the pulmonary vasculature, the finding presented in the current study suggests that thromboxane A_2_ may have the potential to be used as a therapeutic target for pulmonary hypertension due to COPD. This is further supported by the previous findings demonstrating that daltroban, a thromboxane A_2_ receptor antagonist, and celecoxib, a selective cyclooxygenase 2 inhibitor, can reduce cigarette smoke-induced pulmonary artery cell proliferation, likely through the blocking of increased thromboxane A_2_ [5]. Although the levels of thromboxane A2 have not been assessed in all pulmonary artery cell types, including pulmonary artery smooth muscle cells, the finding presented in the current study is consistent with that of a previous study demonstrating increased thromboxane A_2_ in urinary excretions obtained from patients with different groups of pulmonary hypertension, including COPD-associated pulmonary hypertension [8]. This suggests that thromboxane A_2_ may play a key role in the development of pulmonary hypertension due to COPD. Given that drugs targeting the thromboxane A_2_ pathway are not considered in the clinical guidelines as part of the treatment options for all forms of pulmonary hypertension [1], the findings presented in the current study, together with these previous observations [5], strongly suggest that increased thromboxane A_2_ levels in patients with COPD may have detrimental effects, and that blocking these effects may lead to a novel therapeutic target for this fatal disease.

## 5. Limitations

This study has some limitations. Firstly, the sample size is small. However, pulmonary artery cells, particularly pulmonary artery smooth muscle cells, are difficult to obtain, as invasive procedures are required. In addition, pulmonary artery smooth muscle cells grow in a special cell culture medium. Secondly, although pulmonary artery smooth muscle cells were obtained from healthy donors without known respiratory or cardiovascular diseases, as indicated by Thermo Fisher Scientific, Cell Applications and Lonza Group, demographic data and pulmonary function test results are not available. Thirdly, given the fact that our previous study demonstrated that cigarette smoke extract increased thromboxane A_2_, it is highly likely that cigarette smoke, the greatest risk factor for COPD, plays a role in the increased thromboxane A_2_. However, as the levels of thromboxane A_2_ in pulmonary artery smooth muscle cells were not assessed in the current study, further studies assessing thromboxane A_2_ production in smokers without COPD are needed to further confirm the findings presented in the current study. In addition, a controlled trial with a larger sample size is required to further strengthen the findings presented here and to explore the levels of other prostanoids, such as prostaglandin I2 and prostaglandin E2.

## 6. Conclusions

The novel finding that thromboxane A_2_ production is elevated in pulmonary artery smooth muscle cells isolated from smokers with COPD enhances our knowledge of the possible role of thromboxane A_2_ in the development of pulmonary vascular remodelling in COPD and suggests that thromboxane A_2_ may serve as a potential therapeutic target for patients with COPD-associated pulmonary hypertension. Studies are needed to explore the levels and roles of other prostanoids, such as prostaglandin I2 and prostaglandin E2, and their involvement in the development of vascular remodelling in patients with COPD.

## Figures and Tables

**Table 1:** Demographics and clinical characteristics (*n* = 3).

Variable	Mean ± SEM Unless Stated Otherwise
Age	69 ± 7
Male, *n* (%)	3 (100%)
Post-bronchodilator FEV_1_ (L)	1.9 ± 0.57
Post-bronchodilator FEV_1_ (%)	63.33 ± 19.60
Post-bronchodilator FVC (L)	3.79 ± 0.80
Post-bronchodilator FVC/FEV_1_ (%)	52.66 ± 14.64
Smoking history (pack/year)	39.66 ± 9.50

Abbreviations: FEV_1_: forced expiratory volume in one second; FVC: forced vital capacity.

## Data Availability

Data are available upon reasonable request.
